# Routes to psychotic symptoms: Trauma, anxiety and psychosis-like experiences

**DOI:** 10.1016/j.psychres.2008.07.009

**Published:** 2009-09-30

**Authors:** Daniel Freeman, David Fowler

**Affiliations:** a Department of Psychology, Institute of Psychiatry, King's College London, London, UK; b School of Medicine, Health Policy and Practice, University of East Anglia, Norwich, UK

**Keywords:** Delusions, Hallucinations, Psychosis, Trauma, Anxiety

## Abstract

A social factor that has gained recent attention in understanding psychosis is trauma. In the current study the association of a history of trauma with persecutory ideation and verbal hallucinations was tested in the general public. Further, putative mediation variables including anxiety, depression and illicit drug use were examined. In a cross-sectional study, 200 members of the UK general public completed self-report questionnaires. A history of trauma was significantly associated with both persecutory ideation and hallucinations. Severe childhood sexual abuse and non-victimisation events were particularly associated with psychotic-like experiences. The association of trauma and paranoia was explained by levels of anxiety. The association of trauma and hallucinations was not explained by the mediational variables. The study indicates that trauma may impact non-specifically on delusions via affect but that adverse events may work via a different route in the occurrence of hallucinatory experience. These ideas require tests in longitudinal designs.

## 1 Introduction

Psychosis is likely to arise from a number of interacting factors (Van Os and Verdoux, 2003; Garety et al., 2007). One potential factor that has gained some prominence recently is trauma. A number of studies, in both clinical and non-clinical populations, have found an association of trauma and psychotic symptoms (e.g. Bebbington et al., 2004; Read et al., 2005; Scott et al., 2007a; Shevlin et al., 2007). For instance, Janssen and colleagues (2004), in a longitudinal survey of 4000 adults in the general population, found that a history of childhood abuse increased the risk for the subsequent development of psychotic symptoms by over 10 times. This has led to theoretical speculation about how trauma may increase the likelihood of delusions and hallucinations (e.g. Morrison et al., 2003; Fowler et al., 2006a; Spauwen et al., 2006) and a smaller number of empirical studies investigating the ideas (e.g. Kilcommons and Morrison, 2005; Hardy et al., 2005).

The evidence for the association between trauma and psychosis is strongest for the occurrence of hallucinations (Read et al., 2003). If trauma has a causal role, then its influence on delusions and hallucinations may be somewhat different. For instance, the role of trauma in hallucinations may be quite direct, whereby trauma-related intrusions and flashbacks enter the content of hallucinations (e.g. Hardy et al., 2005; Scott et al., 2007b). On the other hand the influence of trauma on paranoia may be non-specific. The experience of trauma is likely to create negative ideas about the self, anxiety, and depression, which are known risk factors for paranoia (e.g. Fowler et al., 2006b; Freeman, 2007). Evidence consistent with these hypotheses was recently found by our research group in a cross-sectional study of 200 students (Gracie et al., 2007).

In this article, we report a replication study, but with a number of significant improvements: the sample studied was broadly representative of the general population for socio-economic status; the intellectual functioning of participants was known; new measures that capture clear persecutory and hallucinatory experiences were used; traumatic events that met a diagnostic criterion for severity were assessed (American Psychiatric Association, 2000); and there were additional measures of depression and anxiety. It was predicted that previous experience of severe traumatic events would be associated with current levels of non-clinical paranoia and hallucinations. Further, it was predicted that experience of trauma would have its effect on paranoia via affect, specifically anxiety and negative beliefs about the self. Finally, a related route by which trauma may influence psychosis was assessed: use of illicit drugs. Both trauma and psychosis are associated with the use of substances such as cannabis (e.g. Kendler et al., 2000; Henquet et al., 2008). It is plausible that coping with the emotional consequences of trauma by taking illicit drugs could contribute to the development of psychotic-like experiences. However, causal claims from a cross-sectional study are clearly limited.

## 2 Method

Two hundred members of the general public completed a trauma assessment and symptom measures as part of a virtual reality study of paranoia (Freeman et al., 2008). The current report concerns unreported tests of associations between the baseline assessments used in the study.

### 2.1 Participants

A representative sample of the UK adult local population was recruited via distribution of a leaflet to 20,000 households in local south London postcodes. The postcodes score highly on indexes of deprivation, consistent with the inner city location. Approximately 350 people responded to the advertisement. The leaflets concerned ‘virtual reality research at King's College London' and did not mention paranoia, hallucinations, trauma or psychiatry. Seven individuals reporting a history of Axis-1 severe mental illness (e.g. schizophrenia, bipolar disorder) were excluded. Two individuals with a history of epilepsy were also excluded because of potential virtual reality side effects (Freeman, 2008). One hundred male and one hundred female participants were recruited. The occupationally based National Statistics Socio-economic Classification was used to categorise participants (Office for National Statistics, 2005). The study had received approval from the research ethics committee of King's College London.

### 2.2 Measures

#### 2.2.1 Trauma

##### 2.2.1.1 Life Stressor Checklist (Wolfe and Kimerling, 1997)

The checklist asks respondents about the occurrence of a range of severe life events (e.g. serious accident, physical attack, sexual abuse). If the respondent reports the occurrence of an event, subsequent questions ask when the event happened, whether the person thought at the time serious harm or death could result, and whether feelings of intense helplessness, fear or horror occurred. Only checklist items that referred to Criterion A events as defined in the Diagnostic and Statistical Manual of Mental Disorders (American Psychiatric Association, 2000) were used. Only events that reached the severity criterion related to post-traumatic stress disorder diagnosis were scored as occurring. The total number of traumatic events, the total number of victimisation events, the number of childhood traumatic events, and the number of traumatic events in the past year were recorded. McHugo and colleagues (2005) report adequate test–retest reliability of the measure in a sample of 200 women.

#### 2.2.2 Paranoia and hallucinations

##### 2.2.2.1 Green et al. Paranoid Thoughts Scale—Part B (Green et al., 2008)

The G-PTS Part B is a trait measure of persecutory ideation. Each of the 16 items (e.g. ‘I was convinced there was a conspiracy against me’, ‘Certain individuals have had it in for me’, ‘I have definitely been persecuted’) conforms to a recent definition of persecutory ideation (Freeman and Garety, 2000) and is rated on a scale from 1 to 5. The presence of persecutory ideation is assessed over the past month and higher scores indicate greater levels of persecutory thinking. The questionnaire has been psychometrically evaluated for use in both clinical and non-clinical populations. The internal consistency of the scale and the test–retest reliability are good. Convergent validity with the Paranoia Scale (Fenigstein and Vanable, 1992) has been shown.

##### 2.2.2.2 Cardiff Anomalous Perceptions Scale (Bell et al., 2006)

This 32-item questionnaire, developed in both non-clinical and psychosis groups, assesses a range of perceptual anomalies. The measure shows good construct and criterion validity, internal reliability and test–retest reliability. In the current study we focussed upon the three verbal hallucinations items together (‘Do you ever hear voices commenting on what you are thinking or doing?’, ‘Do you ever hear voices saying words or sentences when there is no one around that might account for it?’, ‘Have you ever heard two or more unexplained voices talking with each other?’). The scale also has three factor scores. The first factor, temporal lobe experience, contains items such as ‘Do you ever think that everyday things look abnormal to you?’ and ‘Do you ever see shapes, lights, or colors even though there is nothing really there?’. The second factor, chemosensation, contains items such as ‘Do you ever notice that food or drink seems to have an unusual taste?’ and ‘Do you ever smell everyday odors and think that they are unusually strong?. The third factor, clinical psychosis, contains items such as ‘Do you ever hear your own thoughts spoken aloud in your head, so that someone near might be able to hear them?’ and ‘Do you ever hear voices commenting on what you are thinking or doing?’. Respondents initially rate (No/Yes) whether they have experienced the anomaly. A higher score represents the reporting of a greater number of perceptual anomalies.

#### 2.2.3 Intelligence

##### 2.2.3.1 Wechsler Abbreviated Scale of Intelligence (Wechsler, 1999)

The WASI is a nationally standardised short and reliable measure of intelligence linked to the Wechsler Adult Intelligence Scale—Third Edition (Wechsler, 1997). The Vocabulary and Matrix Reasoning subtests were used in the current study.

#### 2.2.4 Affective processes

##### 2.2.4.1 Depression Anxiety Stress Scales (Lovibond and Lovibond, 1995)

The DASS is a 42-item instrument with three subscales measuring current symptoms of depression, anxiety, and stress. Each of the subscales consists of 14 items with a 0–3 scale (0 = did not apply to me at all, 3 = applied to me very much). Higher scores indicate higher levels of emotional distress. The scale has been shown to be reliable and valid in a large UK non-clinical population (Crawford and Henry, 2003). The anxiety and depression subscales were used in the current study.

##### 2.2.4.2 Brief Core Schema Scales (Fowler et al., 2006a,b)

This measure, developed with non-clinical and psychosis groups, has 24 items each rated on a 5-point scale (0–4). Four subscale scores are derived: negative beliefs about self, positive beliefs about self, negative beliefs about others and positive beliefs about others. Higher scores reflect greater endorsement of items. In this study the scale Negative beliefs about self was of interest.

#### 2.2.5 Illicit drug use

##### 2.2.5.1 Maudsley Addiction Profile (Marsden et al., 1998)

The MAP was developed with a large sample from a substance abuse clinic. Respondents are asked directly about the use over the past month of illicit drugs including cannabis, cocaine powder, crack cocaine, heroin, amphetamines, and methadone. Use of illicit drugs was dichotomised into absence and occurrence (meaning that any level of drug use in the past month would count as occurrence).

### 2.3 Analysis

All analyses were carried out using SPSS Version 15.0 (2006). All hypothesis testing was two-tailed, and 95% confidence intervals (CI) are reported. The key variables of history of trauma, paranoid ideation, and verbal hallucinations were dichotomised (presence/absence) and relationships between these variables were then assessed with Chi-square tests. (As a check that the associations were not simply determined by demographic differences, binary logistic regressions controlling for age, sex, ethnicity, education level, socio-economic status, and intellectual functioning were then carried out.) The variables anxiety, depression, negative ideas about the self, and illicit drug use were then used to understand the nature of any significant associations between trauma and psychotic-like experiences (Baron and Kenny, 1986). The putative mediation variables were first tested for associations with trauma and symptoms using *t*-tests or Chi-square. Then, two binary logistic regressions were carried out with paranoia and hallucinations as the dependent variables, and lifetime trauma and potential mediator variables as the independent variables. These regressions were also repeated controlling for demographic information.

## 3 Results

### 3.1 Demographic data

The average age of the participants was 37.5 (S.D. = 13.3) (minimum = 18, maximum = 77). The mean IQ score was 104.6 (S.D. = 12.0) (minimum = 69, maximum = 133). Further basic information on the participants is presented in Table 1. There is a spread of participants across socio-economic categories, and the proportion in each category is broadly representative of the United Kingdom population.

### 3.2 Occurrence of traumatic events

At least one traumatic event had been experienced by 70% of the sample (*n* = 140). Childhood physical or sexual abuse had been experienced by 25.5% (*n* = 51), with 7.5% experiencing severe childhood sexual abuse (*n* = 15). A traumatic event had been experienced in the last year by 15%. Endorsement rates for individual events are presented in Table 2.

### 3.3 Occurrence of persecutory ideation and anomalous perceptions

The mean persecutory ideation score was 22.6 (S.D. = 11.6) (range 16–78). Paranoid ideation was not reported by 42.5% of the sample(*n* = 85), and the scores showed an exponential distribution similar to other studies (Freeman et al., 2005). Verbal hallucinations were reported by 15.5% of the sample (*n* = 31), and at least one anomalous experience in the clinical psychosis factor was reported by 44.5%r (*n* = 89). There were higher rates for endorsement of at least one item for the temporal lobe (75%) and chemosensation factors (72%).

### 3.4 Associations of traumatic events and psychotic-like experiences

The occurrence of at least one lifetime traumatic event was associated with a 2.5 times greater risk of endorsing a persecutory thought than not reporting any traumatic event (see Table 3). Similarly, the occurrence of at least one lifetime traumatic event was associated with a 4.8 times greater risk of endorsing a verbal hallucinations item. (These associations of lifetime trauma and psychosis-like experiences remained significant in logistic regressions controlling for age, sex, ethnicity, education level, socio-economic status, and intellectual functioning.) It is also of note that a history of lifetime trauma was associated with higher scores on the Cardiff Anomalous Perception Scale factors of temporal lobe experience, chemosensation, and clinical psychosis (all *P*-values < 0.01).

Type of trauma was also examined (Table 3). Using the whole sample, it can be seen that non-victimisation events and severe childhood sexual abuse were particularly associated with psychotic-like experiences. Recent adult traumas did not have an impact. When the group with no history of any trauma was used as the comparison (*n* = 60), all trauma types, except adult history of physical attack, were significantly associated with the presence of paranoid ideation and verbal hallucinations (*P* < 0.05). The analysis was also repeated removing the individuals without a history of trauma (i.e. looking within the group who had experienced at least one trauma). Experience of non-victimisation events was significantly associated with both paranoid ideation and verbal hallucinations (*P* < 0.05). Severe childhood abuse was strongly associated with verbal hallucinations (*P* = 0.001), and almost had a near-significant association with paranoia (*P* = 0.056). All other comparisons were non-significant (*P* > 0.05).

### 3.5 Mediation analyses

The presence of at least one lifetime trauma was significantly associated with higher levels of anxiety, depression and negative ideas about the self (all *P*-values < 0.05) (see Table 4). Lifetime trauma was also associated with current use of illicit drugs, Chi-square (df = 1) = 4.586, *P* = 0.032, odds ratio = 2.14, CI = 1.06, 4.32. The presence of paranoid ideation was significantly associated with higher levels of anxiety, depression and negative ideas about the self (all *P*-values < 0.01). Persecutory ideation was also associated with current use of illicit drugs, Chi-square (df = 1) = 8.640, *P* = 0.003, odds ratio = 2.57, CI = 1.36, 4.88. The presence of verbal hallucinations was significantly associated with higher levels of anxiety (*P* < 0.05), had a trend to be associated with higher levels of depression (*P* = 0.068) but was not associated with negative beliefs about the self (*P* = 0.122). Verbal hallucinations were not significantly associated with current use of illicit drugs, Chi-square (df = 1) = 0.749, *P* = 0.387, odds ratio = 0.68, CI = 0.29, 1.62. Illicit drug use was associated with higher levels of anxiety (*P* = 0.006), but not significantly with levels of depression (*P* = 0.058) or negative ideas about the self (*P* = 0.237).

Only potential mediation variables that had a significant association with trauma and paranoia or hallucinations were entered into the next stage of the analysis. A binary logistic regression was carried out with paranoid ideation as the dependent variable and a history of trauma, anxiety, depression, negative ideas about the self, and illicit drug use entered as independent variables (see Table 5). Only anxiety was a significant predictor in this model. A similar finding was obtained including age, sex, ethnicity, education, intellectual functioning, and socio-economic status as covariates. A further binary logistic regression was carried out with verbal hallucinations as the dependent variable and a history of trauma and anxiety as independent variables. Both lifetime trauma and anxiety were significant predictors (see Table 5). When age, sex, ethnicity, education, intellectual functioning, and socio-economic status were entered as covariates, then lifetime trauma remained significant (*P* = 0.021), but the association with anxiety became a trend (*P* = 0.055).

## 4 Discussion

The study provides further confirmation that lifetime experience of trauma is common in the general population. Seventy percent of the general population sample had experienced a trauma, although this rate is much higher than the findings from large epidemiological studies (Kessler et al., 1995; Perkonigg et al., 2000; Breslau, 2002), and severe traumas such as childhood sexual abuse were present in a much smaller proportion of the group. However the focus of the current study was not on trauma prevalence but on identifying links between trauma and instances of psychotic-like phenomena. Clear associations were found. Experiencing a trauma was associated with almost a five times greater likelihood of verbal hallucinations and approximately half that risk of paranoid thoughts. These results are consistent with previous research (Campbell and Morrison, 2007; Gracie et al., 2007). It is difficult to disentangle the effect of different trauma types, but in this sample there was evidence that psychotic-like experiences had the strongest associations with severe childhood sexual abuse and, unexpectedly, non-victimisation events.

Trauma may impact in different ways on persecutory thinking and hallucinatory experience. Previous research has provided strong evidence of a link between anxiety and persecutory thinking (Freeman and Freeman, 2008). Anxiety has repeatedly been found to be associated with paranoid thoughts (e.g. Martin and Penn, 2001) and persecutory delusions (e.g. Freeman and Garety, 1999). Further, anxiety is predictive of the occurrence of paranoid thoughts (e.g. Freeman et al., 2008) and of the persistence of persecutory delusions (Startup et al., 2007). Moreover, it has been shown in non-clinical groups that paranoid thoughts build upon common interpersonal anxieties and worries (Freeman et al., 2005). The results of the current study indicate that trauma influences persecutory thinking non-specifically via the creation of anxiety. Anxiety also accounted for the association of substance use and paranoia. Anxiety may be a common linking factor between trauma, illicit drug use, and paranoia. However it is entirely plausible, and supported by the presence of a statistical trend in the mediational analysis, that the role of illicit drug use in paranoia has a degree of independence from anxiety.

In contrast the link between trauma and hallucinations was unexplained by the small number of putative mediational variables assessed in the current study. A history of trauma and levels of anxiety were independently associated with the occurrence of verbal auditory hallucinations. In this study we clearly did not succeed in including variables relevant to the association between trauma and hallucinations. Specific assessments of trauma-related intrusions and flashbacks were needed to have tested hypothesised direct links between trauma and hallucinations (e.g. Morrison et al., 2003; Scott et al., 2007b), although it might be argued that levels of anxiety are a proxy for, or at least highly correlated, with these processes. It would also have been of interest to have included assessments relevant to inner speech and self-monitoring models of voices (e.g. Allen et al., 2007; Jones and Fernyhough, 2007).

There are weaknesses in the study that merit caution in interpreting the results. Foremost, the cross-sectional study design makes both causal and mediation claims weak. There was multiple statistical testing raising the likelihood of the occurrence of Type I errors. It is also unlikely that the sample is truly representative of the UK general population. The reliance on self-report is important to note. This is most problematic for persecutory thinking. It is plausible that there was overlap between responses for the paranoia and trauma questionnaires. That is, a proportion of the persecutory thinking may not have been unfounded and instead related directly to the past trauma. In the experimental study the participants took part in, where unfounded paranoia was assessed, no direct association of trauma and paranoia was identified (Freeman et al., 2008). This highlights the need for the study of trauma and psychosis to go beyond the establishment of cross-sectional self-report associations.

## Figures and Tables

**Table 1:** Demographic data for the sample.

Variable	Number
Sex	
Male	100 (50%)
Female	100 (50%)
Ethnicity	
White	135 (67.5%)
Black Caribbean	18 (9%)
Black African	9 (4.5%)
Black Other	5 (2.5%)
Indian	6 (3%)
Pakistani	1 (0.5%)
Other	26 (13%)
Highest education level achieved	
None	11 (5.5%)
GCSE	39 (19.5%)
AS/A level	30 (15%)
Diploma/Foundation	27 (13.5%)
Degree	55 (27.5%)
Postgraduate diploma	34 (17%)
Doctoral degree	4 (2%)
Socio-economic classification [Study figure, National figure]	
Higher professional occupations	16 [8%, 11.1%]
Lower managerial and professional occupations	57 [28.5%, 22.4%]
Intermediate occupations	17 [8.5%, 10.0%]
Small employers and own account workers	12 [6%, 7.6%]
Lower supervisory and technical occupations	8 [4%, 9.1%]
Semi-routine occupations	17 [8.5%, 12.8%]
Routine occupations	13 [6.5%, 9.3%]
Never worked and long term unemployed	33 [16.5%, 3.8%]
Not classifiable (students)	27 [13.5%, 13.7%]
Mean IQ (SD)	104.6 (12.0)

**Table 2:** Endorsement rates for each traumatic event.

	Endorsed at Criterion A level
*Non-victimisation*	
Have you ever seen a robbery, mugging or attack taking place?	24.5%
When you were young (before age 16), did you ever see violence between family members (for example, hitting, kicking, slapping, punching)?	19%
Have you ever had a very serious physical or mental illness (for example, cancer, heart attack, serious operation, felt like killing yourself, been hospitalised because of nerve problems)?	16.5%
Have you ever seen a serious accident?	16%
Have you ever had a very serious accident or accident-related injury (for example, a bad car wreck or an on-the-job accident)?	13.5%
Has someone close to you died suddenly or unexpectedly (for example, sudden heart attack, murder or suicide)?	10.5%
Have you ever been in a serious disaster?	7.5%
Has someone close to you died (do not include those who died suddenly or unexpectedly)?	4%
Have you ever had an abortion or miscarriage (lost your baby)?	3%

*Victimisation*	
Have you ever been robbed, mugged, or physically attacked (not sexually) by someone you did not know?	22%
Have you ever been emotionally abused or neglected (for example, being frequently shamed, embarrassed, ignored or repeatedly told that you were “no good”)?	10.5%
After age 16, were you ever abused or physically attacked (not sexually) by someone you knew (for example, a parent, boyfriend, or husband hit, slapped, choked, burned, or beat you up)?	11.5%
After age 16, did you ever have sex (oral, anal, genital) when you didn't want to because someone forced you in some way or threatened to harm you if you didn't?	9%
After age 16, were you ever touched or made to touch someone else in a sexual way because he/she forced you in some way or threatened to harm you if you didn't?	6%
Have you ever been physically neglected (for example, not fed, not properly clothed, or left to take care of yourself when you were too young or ill)?	4%
Have you ever been bothered or harassed by sexual remarks, jokes, or demands for sexual favors by someone at work or school (for example, a coworker, a boss, a customer, another student, a teacher)?	1%

*Childhood physical and sexual abuse (also included within victimisation)*	
Before age 16, were you ever touched or made to touch someone else in a sexual way because he/she forced you in some way or threatened to harm you if you didn't?	16.5%
Before age 16, were you ever abused or physically attacked (not sexually) by someone you knew (for example, a parent, boyfriend, or husband, hit, slapped, choked, burned, or beat you up)?	13.5%
Before age 16, did you ever have sex (oral, anal, genital) when you didn't want to because someone forced you in some way or threatened to harm you if you didn't?	7.5%

**Table 3:** Cross-tabulation of history of trauma with psychotic-like experiences.

Trauma type	Paranoid ideation		Verbal hallucinations	
	No	Yes	No	Yes
No history of trauma (*n*)	35 (58%)	25 (42%)	57 (95%)	3 (5%)
History of trauma (*n*)	50 (36%)	90 (64%)	112 (80%)	28 (20%)
	*χ*^2^ (df = 1) = 8.79, *P* = 0.003**, odds ratio = 2.52, CI = 1.36, 4.68.		χ^2^ (df = 1) = 7.22, *P* = 0.007**, odds ratio = 4.75, CI = 1.39, 16.29	
No non-victimisation (*n*)	50 (58%)	36 (42%)	82 (95%)	4 (5%)
Non-victimisation event (*n*)	35 (31%)	79 (69%)	87 (76%)	27 (24%)
	*χ*^2^ (df = 1) = 15.10, *P* = < 0.001***, odds ratio = 3.14, CI = 1.75, 5.63.		*χ*^2^ (df = 1) = 13.56, *P* < 0.001***, odds ratio = 6.36, CI = 2.13, 18.97.	
No childhood abuse (*n*)	69 (46%)	80 (54%)	131 (88%)	18 (12%)
Childhood abuse (*n*)	16 (31%)	35 (69%)	38 (75%)	13 (25%)
	*χ*^2^ (df = 1) = 3.47, *P* = 0.063, odds ratio = 1.89, CI = 0.96, 3.70.		*χ*^2^ (df = 1) = 5.22, *P* = 0.022*, odds ratio = 2.49, CI = 1.112, 5.54.	
No victimisation (*n*)	51 (50%)	51 (50%)	87 (85%)	15 (15%)
Victimisation (*n*)	34 (35%)	64 (65%)	82 (84%)	16 (16%)
	*χ*^2^ (df = 1) = 4.79, *P* = 0.029*, odds ratio = 1.88, CI = 1.06, 3.33.		*χ*^2^ (df = 1) = 0.10, *P* = 0.752, odds ratio = 1.13, CI = 0.53, 2.44.	
No recent trauma (*n*)	77 (45%)	93 (55%)	145 (85%)	25 (15%)
Recent trauma (*n*)	8 (27%)	22 (73%)	24 (80%)	6 (20%)
	*χ*^2^ (df = 1) = 3.62, *P* = 0.057, odds ratio = 2.28, CI = 0.96, 5.40.		*χ*^2^ (df = 1) = 0.55, *P* = 0.460, odds ratio = 1.45, CI = 0.54, 3.90.	
No childhood unwanted sex (*n*)	83 (45%)	102 (55%)	162 (88%)	23 (12%)
Childhood unwanted sex (*n*)	2 (13%)	13 (87%)	7 (47%)	8 (53%)
	*χ*^2^ (df = 1) = 5.65, *P* = 0.018*, odds ratio = 5.29, CI = 1.16, 24.10.		*χ*^2^ (df = 1) = 17.72, *P* < 0.001***, odds ratio = 8.05, CI = 2.67, 24.29.	
No adulthood unwanted sex (*n*)	81 (45%)	101 (55%)	154 (85%)	28 (15%)
Adulthood unwanted sex (*n*)	4 (22%)	14 (78%)	15 (83%)	3 (17%)
	*χ*^2^ (df = 1) = 3.33, *P* = 0.068, odds ratio = 2.81, CI = 0.89, 8.86.		*χ*^2^ (df = 1) = 0.02, *P* = 0.886, odds ratio = 1.10, CI = 0.30, 4.05.	
No childhood physical attack (*n*)	78 (45%)	95 (55%)	150 (87%)	23 (13%)
Childhood physical attack (*n*)	7 (26%)	20 (74%)	19 (70%)	8 (30%)
	*χ*^2^ (df = 1) = 3.51, *P* = 0.061, odds ratio = 2.35, CI = 0.94, 5.84.		*χ*^2^ (df = 1) = 4.76, *P* = 0.029*, odds ratio = 2.75, CI = 1.08, 7.00.	
No adulthood physical attack (*n*)	78 (44%)	99 (56%)	152 (86%)	25 (14%)
Adulthood physical attack (*n*)	7 (30%)	16 (70%)	17 (74%)	6 (26%)
	*χ*^2^ (df = 1) = 1.55, *P* = 0.213, odds ratio = 1.80, CI = 0.71, 4.59.		*χ*^2^ (df = 1) = 2.22, *P* = 0.136, odds ratio = 2.15, CI = 0.77, 5.97.	

Percentages provided for rows. * *P* < 0.05, ** *P* < 0.01, *** *P* < 0.001.

**Table 4:** Levels of affect by the presence of paranoia, hallucinations, a history of trauma, and use of illicit drugs.

	Anxiety mean (S.D.)	Depression mean (S.D.)	Negative self mean (S.D.)
No paranoid ideation (*n* = 85)	2.4 (6.1)	4.6 (7.3)	1.7 (2.5)
Paranoid ideation (*n* = 115)	6.1 (5.8)***	9.1 (8.9)***	2.8 (3.0)**
No verbal hallucinations (*n* = 169)	4.1 (4.6)	6.7 (8.3)	2.2 (2.8)
Verbal hallucinations (*n* = 31)	7.0 (6.8)*	9.8 (9.7)	3.1 (3.3)
No lifetime trauma (*n* = 60)	2.8 (3.1)	5.2 (7.6)	1.7 (2.5)
Lifetime trauma (*n* = 140)	5.3 (5.6)***	8.0 (8.8)*	2.6 (3.0)*
No recent illicit drug use (*n* = 135)	3.8 (4.3)	6.4 (8.1)	2.2 (2.9)
Illicit drug use (*n* = 65)	6.2 (6.3)**	8.8 (9.3)	2.7 (2.8)

* *P* < 0.05, ** *P* < 0.01, *** *P* < 0.001.

**Table 5:** Binary logistic regressions with paranoia and hallucinations as dependent variables.

	*B*	SE	*P*-value	Odds ratio	L.C.I.	U.C.I
*Dependent variable: paranoia*						
Lifetime trauma	0.550	0.343	0.108	1.73	0.89	3.40
Anxiety	0.172	0.051	0.001**	1.19	1.08	1.31
Depression	0.032	0.026	0.223	1.03	0.98	1.09
Negative self	0.015	0.072	0.832	1.02	0.88	1.17
Illicit drug use	0.645	0.353	0.068	1.91	0.95	3.81

*Dependent variable: hallucinations*						
Lifetime trauma	1.358	0.639	0.034*	3.89	1.11	13.59
Anxiety	0.073	0.034	0.032*	1.08	1.01	1.15

* *P* < 0.05, ** *P* < 0.01.
